# A family-based satisfaction survey on the ICU

**DOI:** 10.1186/cc11100

**Published:** 2012-03-20

**Authors:** D Moult, R Breeze, A Molokhia

**Affiliations:** 1 University Hospital Lewisham, London, UK

## Introduction

We conducted a prospective survey to determine satisfaction amongst relatives of patients on our ICU. Patient-reported outcome measures have become widely used and accepted in the pursuit of improved quality of care [1]. However, assessing patient satisfaction is difficult on the ICU, an environment where we more commonly communicate with the family of patients regarding the care of their relative. Therefore, a more family-centred approach is indicated, for which family satisfaction questionnaires have already been validated [2].

## Methods

We utilised a 35-point questionnaire-based survey of relatives of patients in our ICU over 10 weeks. Questionnaires were distributed to family members when the decision to discharge from the ICU was made. We limited this to two family members per patient who were in the ICU for more than 48 hours.

## Results

We received 29 completed questionnaires. Relatives of 24 of the respondents had survived to ICU discharge. Responses were linearly transformed to give percentage scores. Higher values represented a greater degree of satisfaction. Overall care in the ICU, 88.8%. Courtesy, respect and compassion to the patient (93.8%), to relatives (92.2%); assessment and treatment of pain (94.4%), breathlessness (92.9%), agitation (88.9%); emotional support (89.4%); care from nurses (92.0%), doctors (95.5%); frequency of communication nurses (92.9%), doctors (89.7%). Overall decision-making, 91.3%. Willingness of staff to answer questions (90.5%); honesty (90.5%), completeness (91.4%), consistency of information (90.5%); inclusion in decision-making, 78.7%; support during decision-making, 78.7%; time to think about information given, 96.2%.

## Conclusion

Family satisfaction with our ICU is high, with satisfaction high in both care and decision-making domains. Appropriate inclusion with and support during the decision-making process were areas with lower satisfaction scores. The structuring of options for answering these questions may have been a confounding factor in this finding. However, this may represent genuine lower levels of satisfaction and steps should be taken to improve this. In response to these findings we have invited families to join their relatives' part of the consultant ward round to improve inclusion and support in decision-making. We are currently repeating the survey with these changes in place and will present our findings in the future.
